# Subcutaneous abdominal wall involvement in diffuse large B-cell lymphoma: case report

**DOI:** 10.3389/fonc.2026.1792763

**Published:** 2026-03-17

**Authors:** Duanyang Xu, Binghan Guo, Manxi Li, Yahan Zhang

**Affiliations:** Department of Ultrasound, The Second Affiliated Hospital of Dalian Medical University, Dalian, China

**Keywords:** abdominal wall, diffuse large B-cell lymphoma, immunohistochemistry, subcutaneous lymphoma, ultrasound

## Abstract

Diffuse large B-cell lymphoma (DLBCL) is the most common subtype of non-Hodgkin lymphoma (NHL) and represents the most frequent malignant lymphoma in adults. It typically occurs in sites such as the neck, axilla, and groin; however, lymphoma originating in the abdominal wall subcutaneous tissue is relatively rare. This case report aims to present the imaging findings and immunohistochemical results of a patient with subcutaneous lymphoma located in the abdominal wall. To our knowledge, this represents a relatively rare case.

## 1 Introduction

Diffuse large B-cell lymphoma is an aggressive tumor derived from mature B cells, characterized by extensive infiltration that can involve multiple lymph nodes and extranodal organs (1, 2). It commonly presents as painless, progressive enlargement of cervical or abdominal lymph nodes. Approximately 30% of patients exhibit extranodal involvement, most frequently affecting the gastrointestinal tract, bones, testes, and other sites (3). It is noteworthy that subcutaneous lymphoma of the abdominal wall is extremely rare. In such cases, the patients typically show no abnormal abdominal protrusion or cutaneous changes, distinguishing it from primary cutaneous lymphomas. This article reports a case of DLBCL presenting as a subcutaneous lymphoid mass in the abdominal wall, aiming to enhance clinicians’ awareness of atypical lymphoma presentations and facilitate early diagnosis and precise treatment, thereby improving patient prognosis.

## 2 Case presentation

A 63-year-old male patient presented to our hospital’s emergency department with a 5-month history of paroxysmal wheezing, which had worsened over the past week accompanied by chest tightness and shortness of breath. The patient reported fever with a peak temperature of 39.0 °C days prior and had experienced significant weight loss of over 5 kg in the past 3 months.

**Figure 1:**
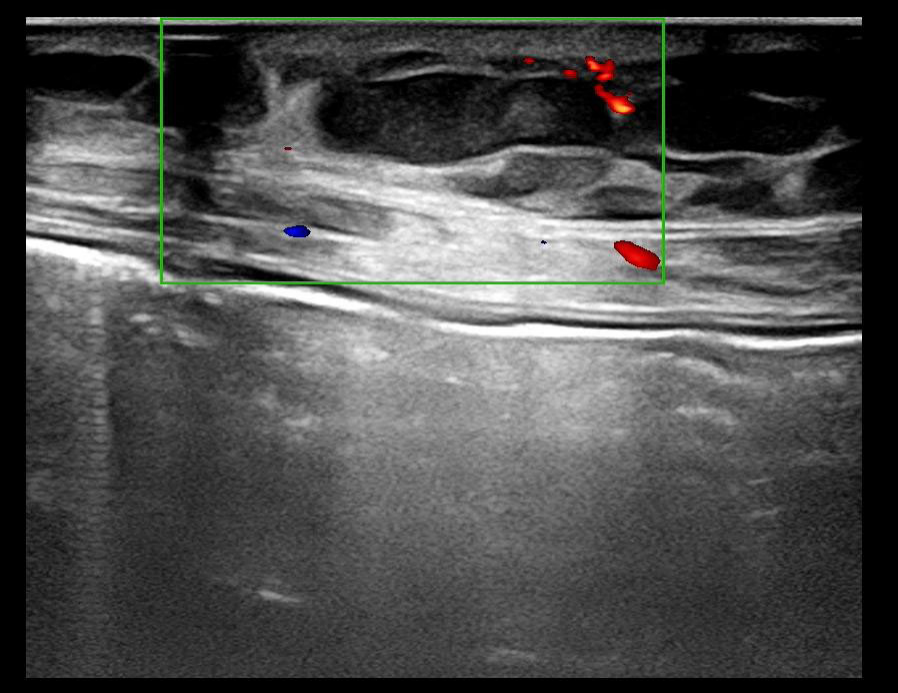
Ultrasound reveals multiple hypoechoic lesions in the abdominal subcutaneous tissue, some of which are confluent, with no blood flow signals detected.

Based on the clinical requirements, a CT scan of the chest and abdomen was performed on the patient. The results revealed multiple nodules within the bilateral subcutaneous tissue of the abdominal wall, accompanied by surrounding exudation. Subsequently, an ultrasonography of the subcutaneous nodules in the abdominal wall was conducted. The ultrasound findings showed multiple hypoechoic lesions, with the largest measuring 1.3 × 0.7 cm and with some demonstrating fusion, clear boundaries, and no detectable blood flow signal (**Figure 1**). The blood tests indicated significantly decreased hemoglobin levels, thrombocytopenia, and a markedly reduced absolute lymphocyte count.

**Figure 2:**
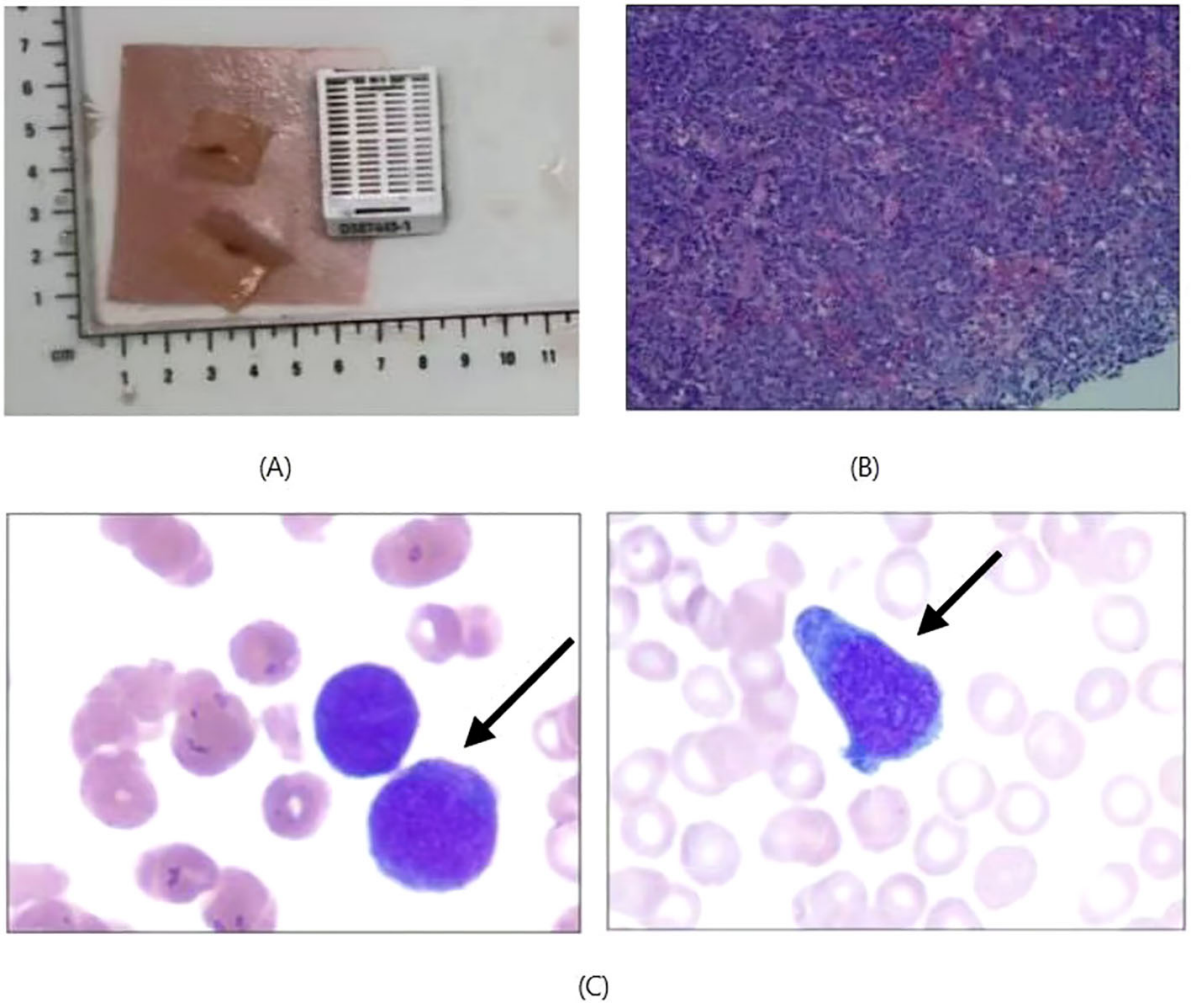
**(A)** Gross specimen of the abdominal lymphoid mass. **(B)** Histopathological section stained with hematoxylin and eosin (H&E), revealing dense infiltration of lymphocytes. The infiltrate forms vague nodular or diffuse growth patterns, with nodules of varying sizes, distinct from the structure of normal lymph nodes. The infiltrating cells are predominantly small- to medium-sized lymphocytes with irregular nuclear contours and dense chromatin. Scattered large cells are visible, featuring prominent nucleoli and easily identifiable mitotic figures. In some areas, a “starry sky” pattern is observed (scattered macrophages phagocytosing nuclear debris), indicating high proliferative activity. **(C)** Bone marrow cytology revealing large cells with abundant cytoplasm. The left panel demonstrates morphological features consistent with type III atypical lymphocytes or lymphoblasts, while the right panel exhibits the typical characteristics of type II atypical lymphocytes.

For a definitive diagnosis, ultrasound-guided fine-needle aspiration biopsy was performed on the lymphoma-like mass in the abdominal wall. Due to the superficial location of the subcutaneous lesion, the ultrasound result clearly displays its boundaries, size, and relationship with surrounding tissues. It also enables real-time guidance of the needle path, avoiding major blood vessels and vital organs, thereby reducing the risk of complications. The immunohistochemical results were as follows: tumor cells positive for CD20 (+) and CD19 (+); negative for CD5 (–); CD10 (10%+), BCL6 (90%+), MUM1 (90%+), C-MYC (10%+), BCL2 (90%+), CyclinD1 (-); CD21 (no FDC networks observed); p53 (10% weakly +); CD30 (-), ALK (-); Ki67 (90%+). In terms of *in situ* hybridization (ISH), the Epstein–Barr virus-encoded RNA (EBER) was negative. The bone marrow biopsy showed no observed significant lymphoid hyperplasia or infiltration by malignant cells. As for the bone marrow cytology, the examination revealed a reduced white blood cell count with a decreased proportion of granulocytes. The lymphocyte population was within normal range, though some lymphocytes exhibited abnormal cytoplasmic morphology and smaller-than-normal size. The proportion of monocytes was increased. The findings were highly suggestive of bone marrow involvement by non-Hodgkin lymphoma. Based on the histomorphology and immunohistochemical profile, a diagnosis of diffuse large B-cell lymphoma (DLBCL), not otherwise specified (NOS), non-germinal center B-cell subtype (according to the Hans algorithm) was established (**Figure 2**).

During hospitalization, the patient’s condition deteriorated, with persistent fever and continuously declining levels of hemoglobin, absolute lymphocyte count, and platelets. However, due to economic constraints, the patient declined further treatment and requested to be discharged home. Follow-up after discharge revealed that the patient’s survival was less than 6 months.

## 3 Discussion

Diffuse large B-cell lymphoma (DLBCL) is the most common malignant lymphoma in adults and a subtype of non-Hodgkin lymphoma. It typically presents with painless, progressive lymphadenopathy and systemic symptoms. Diagnosis relies entirely on pathological biopsy, with masses most commonly found in the lymph nodes of the neck, axilla, or groin. Its characteristic ultrasound features include round, markedly hypoechoic masses with loss of the fatty hilum, abundant and disordered blood flow signals, partially reticular changes, and possible calcification.

Lymphomatous masses in the abdominal wall subcutaneous tissue are relatively rare and represent an extranodal manifestation of DLBCL. The underlying cause is likely malignant clonal proliferation of B cells, with tumor cells reaching the subcutaneous abdominal tissue and proliferating to form masses via hematogenous spread (primary route), direct invasion, or lymphatic dissemination. In this case, imaging revealed multiple hypoechoic lesions in the abdominal subcutaneous tissue, the largest measuring 1.3 × 0.7 cm and with some lesions showing fusion, clear boundaries, and no detectable blood flow signals. Based on clinical symptoms such as fever and weight loss, lymphoma was suspected and later confirmed as DLBCL through pathological examination.

During hospitalization, the patient exhibited a persistent decline in all three blood cell lineages, indicating myelosuppression and suggesting a poor prognosis. The patient’s short survival further confirms this conclusion.

It is also noteworthy that, despite the location of the lymphoma mass in the abdominal subcutaneous tissue, the clinical presentation differed from that of primary cutaneous lymphoma. Localized cutaneous lymphoma often presents as nodules protruding from the skin surface, usually smooth and appearing flesh-colored, reddish, yellowish-brown, or purple. Diffuse cutaneous lymphoma, more common in middle-aged adults, manifests as miliary papules or large, firm nodules (4). In this case, the patient’s abdominal skin appeared smooth and flat without any abnormal signs. Compared with the current case, extranodal soft tissue diffuse large B-cell lymphoma frequently involves multiple muscles and soft tissues, presenting as a soft tissue infection or inflammation. However, it is unresponsive to antibiotic therapy and necessitates a soft tissue biopsy for definitive diagnosis (5).

## 4 Conclusion

In summary, we report a rare case of diffuse large B-cell lymphoma and integrate the patient’s clinical symptoms, imaging findings, and laboratory results. This report aims to alert clinicians that in unusual circumstances, such as unexplained lymphomatous masses in the abdominal wall subcutaneous tissue or other atypical sites for lymphoma, the possibility of lymphoma should also be considered, thereby providing additional diagnostic reference for clinicians.

## Data Availability

The datasets presented in this study can be found in online repositories. The names of the repository/repositories and accession number(s) can be found in the article/supplementary material.
